# A Supplementary System for a Brain-Machine Interface Based on Jaw Artifacts for the Bidimensional Control of a Robotic Arm

**DOI:** 10.1371/journal.pone.0112352

**Published:** 2014-11-12

**Authors:** Álvaro Costa, Enrique Hortal, Eduardo Iáñez, José M. Azorín

**Affiliations:** Brain-Machine Interface Systems Lab, Miguel Hernández University, Elche, Spain; Duke University, United States of America

## Abstract

Non-invasive Brain-Machine Interfaces (BMIs) are being used more and more these days to design systems focused on helping people with motor disabilities. Spontaneous BMIs translate user's brain signals into commands to control devices. On these systems, by and large, 2 different mental tasks can be detected with enough accuracy. However, a large training time is required and the system needs to be adjusted on each session. This paper presents a supplementary system that employs BMI sensors, allowing the use of 2 systems (the BMI system and the supplementary system) with the same data acquisition device. This supplementary system is designed to control a robotic arm in two dimensions using electromyographical (EMG) signals extracted from the electroencephalographical (EEG) recordings. These signals are voluntarily produced by users clenching their jaws. EEG signals (with EMG contributions) were registered and analyzed to obtain the electrodes and the range of frequencies which provide the best classification results for 5 different clenching tasks. A training stage, based on the 2-dimensional control of a cursor, was designed and used by the volunteers to get used to this control. Afterwards, the control was extrapolated to a robotic arm in a 2-dimensional workspace. Although the training performed by volunteers requires 70 minutes, the final results suggest that in a shorter period of time (45 min), users should be able to control the robotic arm in 2 dimensions with their jaws. The designed system is compared with a similar 2-dimensional system based on spontaneous BMIs, and our system shows faster and more accurate performance. This is due to the nature of the control signals. Brain potentials are much more difficult to control than the electromyographical signals produced by jaw clenches. Additionally, the presented system also shows an improvement in the results compared with an electrooculographic system in a similar environment.

## Introduction

In our society there is an increasing concern about helping and assisting people who suffer from motor disabilities. Emerging from this concern, each day, different areas of research are focusing their efforts on developing Human-Machine systems to help people suffering from these conditions [1], [2]. Brain-Machine Interfaces (BMIs) are a clear example of these systems. Depending on the nature of the neural phenomenons analyzed, these systems can be classified as evoked or spontaneous. On the one hand, spontaneous BMIs study those brainwaves that can be voluntarily controlled by a subject. To achieve this control it is usually necessary to have a training period during which the users learn how to control their brain potentials. On the other hand, evoked BMIs rely on the analysis of brain potentials that cannot be controlled by the users. These potentials appear in response to a external stimulus like flashlights or sounds among others [3], [4]. Spontaneous systems are usually focused on generating commands to control a device taking advantage of the users capability to control their EEG signals [5]–[7]. Regarding evoked systems, there are studies focused on generating control commands [8], [9] and also on the evaluation of the brain response to different external stimulus with diagnosis purposes [10]–[12]. Besides, BMIs (both spontaneous and evoked) are used on other topics in the field of human health, such as the measurement of the mental state of a patient (workload, attention level, emotional state,...) [13] or as support systems on rehabilitation processes [14].

BMI systems can also be divided into two big groups depending on the invasion level needed to register signals. Invasive BMI systems register signals directly from the brain using electrodes implanted inside the cortex [15], [16]. This method provides an excellent signal to noise ratio because the electrodes used are placed much closer to the source of the electrical signals. However, their use is limited due to the risk and ethical questions associated to the surgery needed to implant the electrodes under the scalp. On the other hand, for non-invasive BMIs, surgery is not needed. Instead, a set of electrodes is placed over the scalp in order to register the EEG signals. Nowadays, there are many studies focused on helping people with motor diseases based on non-invasive BMI systems, like [17]–[19]. The main disadvantage of non-invasive BMI systems is the quality of the registered signals. Due to volume conduction which is defined as the property, associated to biological tissues, of transmiting electric and magnetic fields from an electric primary source current, the scalp filters electric signals from the brain and there is mixing of signals from different sources. This makes difficult to isolate the signals produced in a single brain area. Due to the localization of those electrodes, EEG signals are also contaminated by several noise sources produced by other physiological factors like blood pressure, skin tension, muscular and ocular movements, etc. All of these unwanted signals are considered artifacts when the goal of the study is to evaluate how EEG signals behave. For that reason, on non-invasive systems, the signal to noise ratio is a critical factor and detecting and filtering artifacts is a fundamental part of the data analysis [20]–[22].

**Figure 1:**
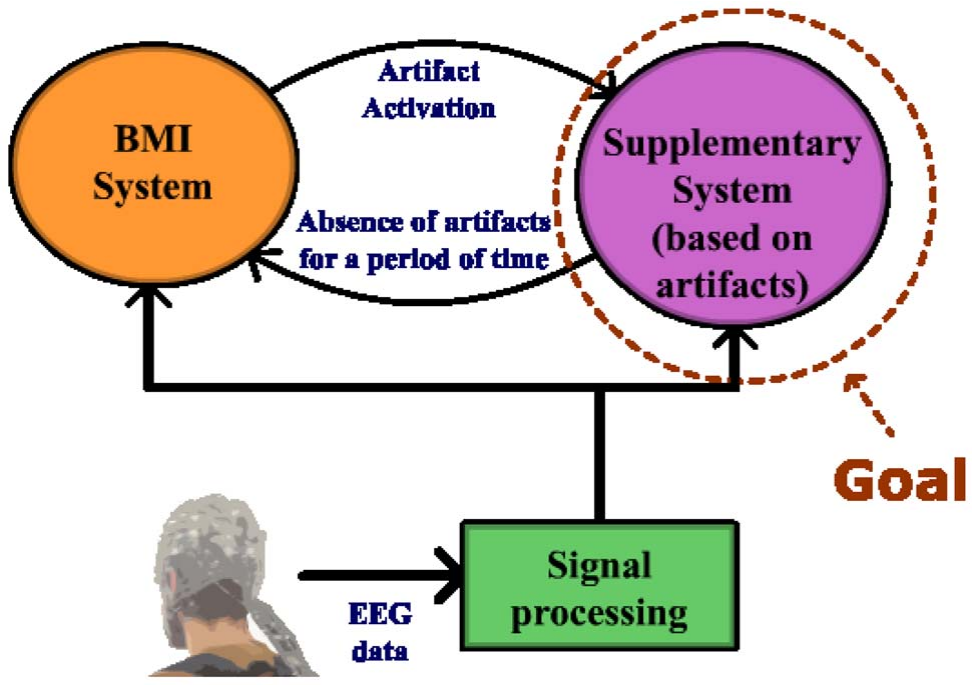
Block interconnection diagram. EEG data from the scalp is acquired, processed and classified in real time. The classification results are used as control commands for 2 different systems. The Supplementary System block is the main goal of the present work. It should be activated and controlled with EMG signals registered from a BMI set of electrodes. If no EMG signals are detected in the EEG data, the BMI system is used. When the user wants to use the Supplementary System, he/she has to generate an EMG signal.

Some signals (usually considered artifacts on BMI systems) can be controlled by users, like those produced by voluntary movements of the eyes (electrooculographic (EOG)) and muscles (electromyographic (EMG)). In this work, the use of EMG signals generated voluntarily by subjects is proposed in order to implement a supplementary system for a BMI. The architecture that appears in Figure 1 shows how this system and the BMI system will coexist. The main advantage of this architecture is that they share the same set of EEG electrodes, instead of including EMG electrodes or other sensors.

The supplementary system proposed uses EMG signals (extracted from the EEG signals of the BMI), which are generated by the users clenching their jaws, in order to control the 2-dimensional movement of a robotic arm. These clench signals affect a wide range of frequencies (1–128 Hz) according to [23]. For that reason is not possible to use this system simultaneously with a BMI system. However, it is possible to freely alternate between both systems (non-simultaneous control). The proposed system, controlled voluntarily by the user as in a spontaneous BMI, has a decreased training time, improved classifier stability and accuracy, and an increased number of the detected tasks. Since this system allows users a better control of a device, it could be used as a complement for a BMI focused on solving other problems also related with the improvement of the quality of life of people with disabilities. For example, internet browsers based on evoked potentials [24] can be complemented with the supplementary system described on this article. Thus the evoked BMI could be used to write text in the browser, while our system could be used to control the cursor.

## Materials and Methods

### Data Acquisition

**Figure 2:**
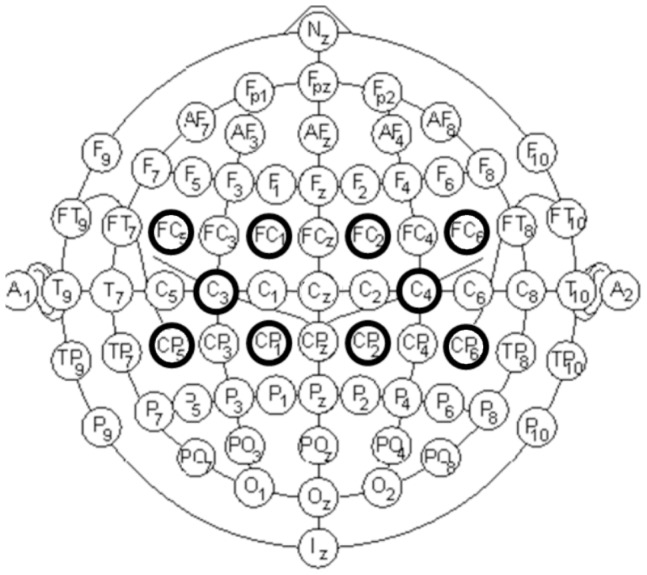
Electrode distribution. FC5, FC1, FC2, FC6, C3, C4, CP5, CP1, CP2 and CP6 (darker circles) according to the International 10/10 System.

**Figure 3:**
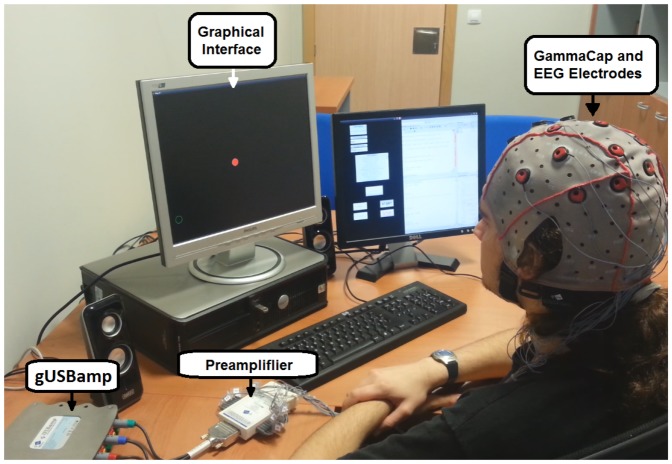
Equipment. Amplifier and GammaCap from g.Tec are used to register EEG Signals. Data are processed and classified in the computer, which is also used to provide visual feedback to the user.

EEG signals are acquired using an amplifier (g.USBamp, g.Tec, GmbH, Austria) with active electrodes to increase their signal/noise ratio by introducing a pre-amplification stage (g.GAMMAbox, g.Tec, GmbH, Austria). The acquisition of EEG signals is done using 10 electrodes placed over the scalp with the following distribution: FC5, FC1, FC2, FC6, C3, C4, CP5, CP1, CP2 and CP6 (see Figure 2) according to the International 10/10 System, with a monoauricular reference in the right earlobe and ground in AFz. Information is digitalized at 1200 Hz. A bandpass filter from 0.1 to 100 Hz has been applied. Also, a 50 Hz Notch filter to remove the power line interference is used. Finally, all the data are sent to a computer system where the processing and classification algorithms are applied. Figure 3 shows an image of the equipment used for the EEG recordings. Electrodes C3 and C4 are the main goal of our analysis. Their readings show the best classification results compared to the other electrodes analyzed. These electrodes are also associated with the sensorimotor areas where right and left motor imagery tasks are detected. The other electrodes shown in Figure 2 (FC5, FC1, FC2, FC6, CP5, CP1, CP2, CP6) are used in the processing stage to remove their power contribution from electrodes C3 and C4.

### Data analysis

The signals are processed in real time as in [25]. To do that, the time between data windows must be small enough for the algorithm to provide feedback to the user in real time. Also, the window length should not be too long to avoid delays in the feedback. Every 50 ms a data window of 400 ms is stored, resulting in a 350 ms overlap between the windows. This overlap increases the stability of the classification results by using the information of previous data windows. Then, a four nearest neighbor Laplacian algorithm [26] is applied to the temporal data from electrodes C3 and C4. This algorithm uses the information received from the four nearest electrodes of C3 and C4 and their distances from them in order to reduce the unwanted signal contribution that these electrodes have on C3 and C4. The result is a smoother time signal where the main contribution comes from the electrode of interest. The Laplacian is computed according to the formula,
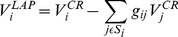
(1)where 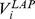 is the result of applying this algorithm to the electrode i, 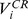 is the electrode i signal before the transformation and, 
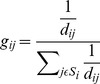
(2)where 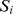 is the set of electrodes that surround electrode i and 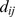 is the distance between i and j electrodes.

**Figure 4:**
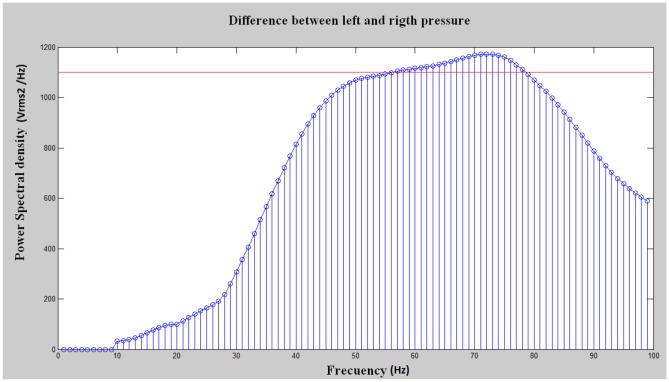
Frequency analysis results. The blue line represent the average Power Spectral Density(PSD) difference between electrodes C3 and C4 for each frequency. The red line represents the minimum spectral level required for a frequency to be target of analysis. These results were obtained from the user 2 (system developer) and compared with the other 3 volunteers to confirm that the optimum range of frequencies does not experience huge changes between users.

Then, the power spectral density of the Laplacian waveforms is computed through the maximum entropy method (MEM) [27]. To differentiate between left and right clenches a frequency analysis is made. For each frequency from 1 to 100 hz, the power spectral density (PSD) of electrodes C3 and C4 is calculated. The difference between these 2 values (C4-C3) is computed when the user clenches the left side of the jaw (C4-C3)L and when the clench is produced on the right side (C4-C3)R. After that, the difference (C4-C3)L - (C4-C3)R is calculated and represented on Figure 4. This initial analysis shows that most differences in the signal power for left and right clenches were present between 57 and 77 Hz and for algorithm only the integral of power spectrum between 57 and 77 Hz was calculated.

### Classification

We have developed a classifier based on the application of different thresholds. First of all, each incoming feature is stored in the first position of a 10 position vector (at first filled with zeros) and the rest of the vector is shifted so the oldest value is lost. Every 50 ms, when a new data window (400 ms length with 350 ms overlap with the previous window) arrives, the average of this vector is compared with four thresholds in order to classify the signals. Five tasks have been established according to the power levels received from this average. Each task is associated with a jaw area and the level of clench pressure, and they are defined as follows:

- HardR: Hard right clench

- SoftR: Soft right clench

- Relax: Not clenching

- SoftL: Soft left clench

- HardL: Hard left clench

**Figure 5:**
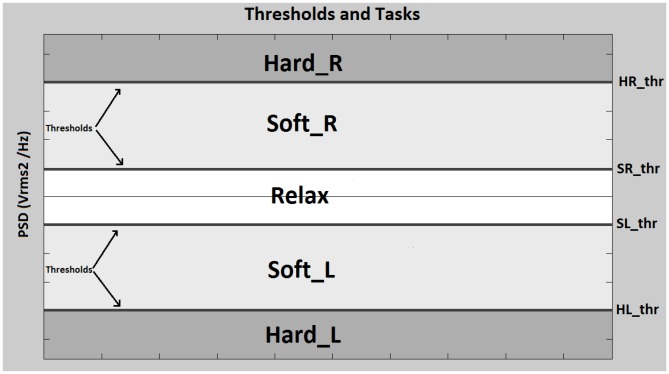
Thresholds and Tasks. Horizontal lines represent the set of thresholds used as model to classify the processed signals. Each threshold represents a level of Power Spectral Density (PSD). Each value on X-axis represents one processed data window. Each data window is classified depending their position on the Y-axis (PSD). Each area delimited by the thresholds represents one of the 5 possible tasks.

**Figure 6:**
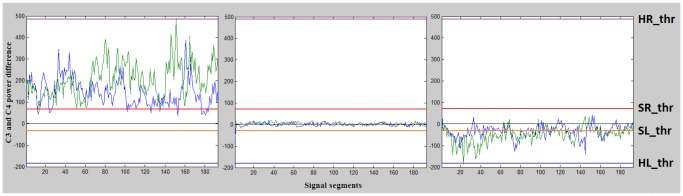
Results obtained for three different tasks during two trials. Signals from the right graph represent two trials (blue and green) where the user clenches the right area of the jaw (SoftR). Signals from the left graph represent two trials where the user clenches the left area of the jaw (SoftL). Signals from the center graph represent two trials where the user releases the jaw (Relax). These signals were recorded from user 2.

These tasks are classified through the comparison of 4 thresholds defined as HRthr, SRthr, SLthr and HLthr. Horizontal lines on Figure 5 show a representation of these thresholds, and the areas represent the 5 possible tasks. In Figure 6, PSD levels of tasks SoftR, Relax and SoftL are shown. The central graph shows two vectors of the classifier input from a subject who is not clenching the jaw (Relax). The right and left graphs display the waveforms caused by clenching right and left jaw areas respectively (SoftR and SoftL). This analysis shows that PSD levels from electrode C3 are higher than PSD levels from C4 when the clenching occurs on the left side of the jaw. In a similar way, C4 levels become dominant when clenching is produced on the right side. On the relax task, the PSD from C3 and C4 are similar so the absolute value of the factor C4-C3 is considerably lower. The absolute value of C4-C3 becomes higher when the clench pressure is higher. If the subjects clench the jaw in such a way that the thresholds HRthr or HLthr are exceeded, tasks HardR and HardL (depending on the jaw side) are detected. If the subjects clench the jaw in such a way that the thresholds SRthr or SLthr are exceeded without reaching HRthr or HLthr, tasks SoftR or SoftL are detected depending, again, on the jaw area where the clench is produced. Finally, if the subject is not clenching his/her jaw, no threshold is exceeded and the relax task is detected.

### User Training

In order to learn how to control the 2-dimensional movement of a cursor on a screen, two graphical interfaces and a three steps training program have been defined. Training steps 1 and 2 use the first graphical interface, while training step 3 uses the second one. Real time data are processed during all three steps and the interfaces provide visual feedback to the users. This way, the users know how the training is progressing in order to improve their results by adapting the way they clench the jaw. In next section, both graphical interfaces are described. The model used through the training is further described on the section Training Steps.

#### Graphical Interfaces

Two graphical interfaces are designed. Both of them show a red cursor with a diameter of 25 pixels (about 1.2 cm with a screen size of 38.5×33 cm). The 2-dimensional movement of this cursor is controlled by the user. Depending on the interface, training step and task, the feedback will be different. This is properly explained on each training step on the section Training Steps.

#### First Interface

**Figure 7:**
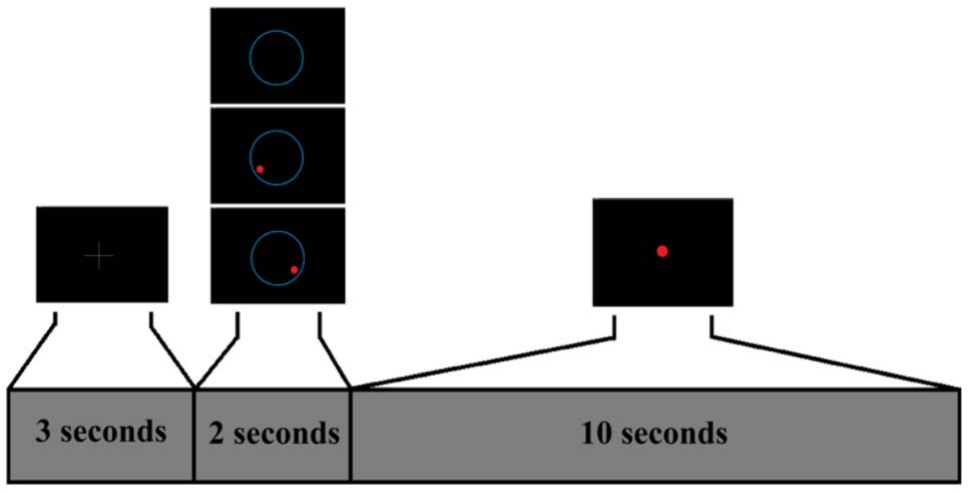
First Interface Protocol. The user is asked to relax during the first 3 seconds, after that, an image appears for 2 seconds to show the task (one of three possible ones) that the user has to perform. For the next 10 seconds, the cursor moves left, right or remains stopped depending on the classified task. After these 10 seconds, the sequence is repeated.

This interface is shown on Figure 7. A white cross is shown for 3 seconds on the screen. During this time, the user rests. Afterwards, an image is shown for 2 seconds to indicate the required task to the user. During these seconds, the user is asked to start performing the task. Finally, a red cursor appears on the screen for 10 seconds. This cursor provides feedback of the task detected by making different movements (in this case only left and right movement are used as feedback, which are further explained in the Training Step section). The control of the cursor depends on the training step. Over one run, this sequence is repeated 15 times making each run 4 minutes long. This interface is used to register data of concrete tasks and create a model adapted to each user.

#### Second Interface

**Figure 8:**
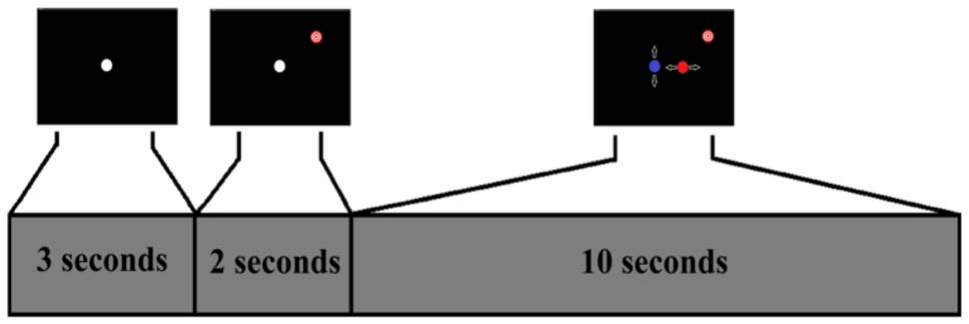
Second Interface Protocol. The user is asked to relax during the first 3 seconds, after that, the user is asked to reach a target. The cursor moves left, right, up, down or remains stopped depending on the classified task until it reaches the target.

Figure 8 shows how the second interface works. First, a white cursor appears on the screen for 3 seconds. After that, a target appears randomly on the screen and both the target and the white cursor remain on the screen for 2 more seconds. Finally, the cursor starts moving depending on the users commands generated by jaw clenches. The main goal of this interface is to simulate the bidimensional movement of the cursor controlled by the user. To do that, the model defined during the training is used. If the user reaches the target, the cursor becomes white and blinks several times as a reward.

#### Training Steps

During these three steps, the user is going to gradually learn how to control the two dimensional cursor movement by clenching the jaw. Each step uses one of the graphical interfaces previously described. All data are processed in real time. From the beginning of the training, the interfaces provide visual feedback to the user by generating cursor movements. In order to produce these movements, a model must exist along the three steps. At first, a default model is defined. During the training, this model is modified to be adapted to the signals of each user.

#### Step 1: Getting used to the clench

The first step starts with the default model (set of thresholds) [10000 100 -100 -10000] (HRthr, SRthr, SLthr and HLthr, respectively from Figure 6). The first graphical interface is used in order to provide visual feedback. The user is asked randomly by the interface to clench softly the right and left jaw areas (tasks SoftR and SoftL). The model is not modified along this step. If the tasks are correctly classified, the red cursor moves right if the clench is produced on the right side, and left if it is produced on the left side. If the clench exceeds thresholds HRthr or HLthr, tasks HardR and HardL are detected and the cursor becomes blue and stops moving. If no threshold is reached, task Relax is detected and the cursor remains red and stopped. During this step, the user gets used to the kind of clench which feels more comfortable with. It has been found that by using this default threshold set, after one 4-minutes run, a user who has never used the system before, is able to control right and left cursor movements. The fact that this default model provides similar results in different users means that the signals do not experience big changes between users.

#### Step 2: Creating the model

**Figure 9:**
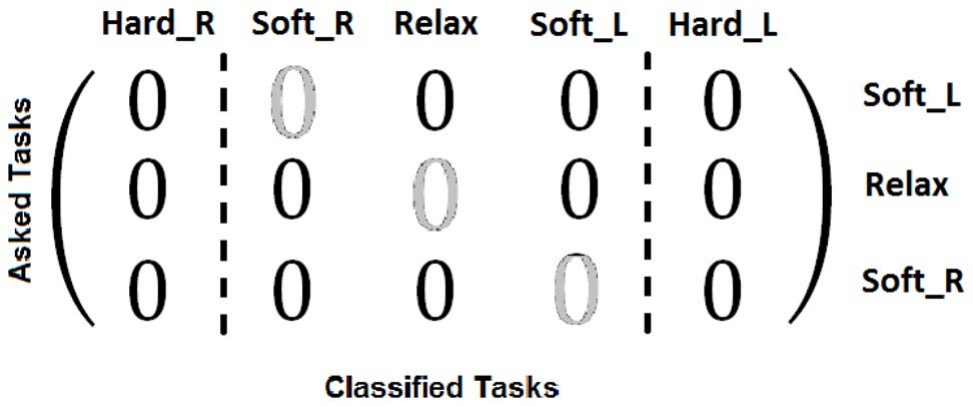
Task classification matrix. Rows represent the tasks requested by the interface and columns represent the tasks classified by the system. Only tasks SoftR, SoftL and Relax are requested while all five tasks (HardR, SoftR, Relax, SoftL and HardL) can be detected. The matrix is initialized with zeros at the beginning of each run and these values are updated with every classification. The thresholds are modified trying to get a diagonal matrix.

**Figure 10:**
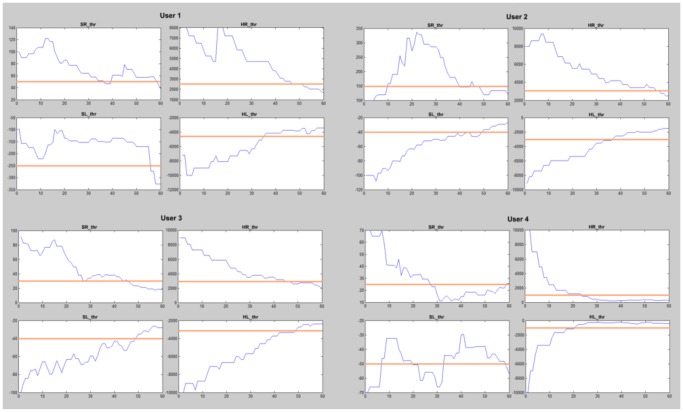
Threshold convergence. The evolution of HRthr, SRthr, SLthr and HLthr are shown for all users. Also the final value selected for each threshold and user is represented with a horizontal line. Y-axis represents the Power Spectral Density and the X-axis represents the number of tasks requested to the user along the 5 runs.

The second step also starts with the default threshold set [10000 100 -100 -10000]. The same interface from step 1 is used but, this time, tasks SoftR, SoftL and Relax are randomly asked. As in step 1, the user has to clench right and left jaw areas softly when tasks SoftR and SoftL are asked (respectively) and keeps the jaw released when the system asks for Relax task. The visual feedback provided by the interface is the same provided in step 1. Tasks SoftR and SoftL move the cursor right and left, respectively, task Relax makes the cursor stop, and tasks HardR and HardL stop the cursor and makes it blue. In this step, after each 10 seconds performed task by the user, a matrix like the one shown in Figure 9 is modified. It is a 3×5 matrix whose rows represent each requested task, while the columns represent the number of classified data windows according to the established set of thresholds (there are three rows because only tasks SoftR, SoftL and Relax are requested while there are five columns because tasks HardR and HardL can be also classified). Each 50 ms a new data window is classified, and the matrix is updated by increasing in one the value of the position indicated by the row requested and the column classified. This way, every 10 seconds it is possible to know which tasks were requested and which tasks were classified by looking at the matrix. According to the amount of wrong classified data windows, thresholds are modified to adjust each task area to the users' skills. If the success rate is 100%, those thresholds that delimit the requested task are reduced by 30%. This way, it is possible to reduce the strength of the clench if the user is able to achieve the same results with softer clenches. Also, reducing the area of the tasks performed could allow the inclusion of new tasks above HardR and below HardL in future works. Otherwise, the thresholds will be increased according to the percentage of misclassified tasks. This process makes threshold values converge to their optimal point. However, this process is endless. Therefore, when thresholds are close to their optimal values, they oscillate around them. After five 4-minutes runs, thresholds reach their oscillation point around their optimal values. The final values are decided by seeing the thresholds evolution and manually selecting them. On Figure 10 this evolution and the final set of thresholds selected are shown for all the users. These final thresholds become the model that best fits the signals produced by each user.

#### Step 3: 2-dimensional movement

**Figure 11:**
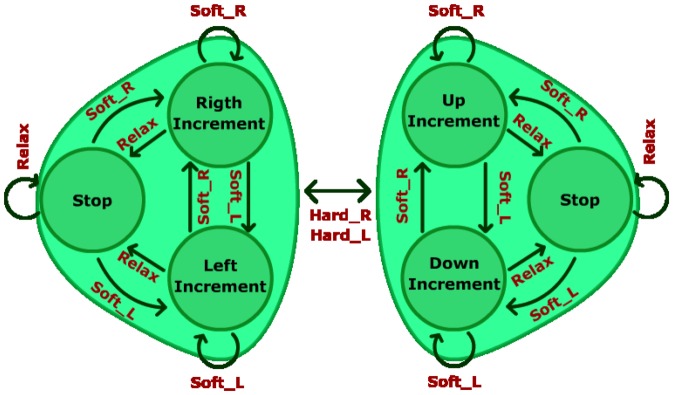
Cursor State Machine. Each group of 3 states represents one of the dimensions. The left group represents the horizontal dimension and the right group represents the vertical dimension. Tasks HardL and HardR are used to alternate between horizontal and vertical dimensions. Once the dimension is selected, tasks SoftR and SoftL control the direction of the movement (on the horizontal dimension, SolfR moves the cursor to the right and SoftL moves the cursor to the left, on the vertical dimension, SoftR moves the cursor up and SoftL moves the cursor down). Relax task stops the cursor no matter the dimension selected.

In the last step, the starting model is the one obtained from step 2 and it does not evolve during this training step. This time, the second interface is used. Eight targets (with the same cursor size) are defined in eight fixed positions and they appear randomly during this step. The user is requested to reach them by controlling the movement of a two dimensional cursor before a time limit is reached. A target is successfully reached when there is less that 15 pixels (0.72 cm) on each axis (X and Y) between the cursor and the target position. Figure 11 shows a state machine that describes the behavior of the cursor depending on the tasks performed by the user. The 2-dimensional axes are not simultaneously controlled by the user but he/she is able to alternate between movement axis using HardR and HardL tasks. This axis alternation is represented by the change of the color of the cursor from red to blue and viceversa. When the cursor turns red, the horizontal dimension is controlled and when it becomes blue, the vertical dimension is controlled. Tasks HardR and HardL are achieved by making a strong clench for a short period of time (less than 0.5 seconds, like a quick bite) on right or left areas of the jaw, respectively. The cursor movement is controlled by tasks SoftR and SoftL (right and left, or up and down according to the current selected axis, respectively). Tasks SoftR and SoftL are achieved by clenching softly the respective area of the jaw. Task Relax stops the cursor movement.

**Figure 12:**
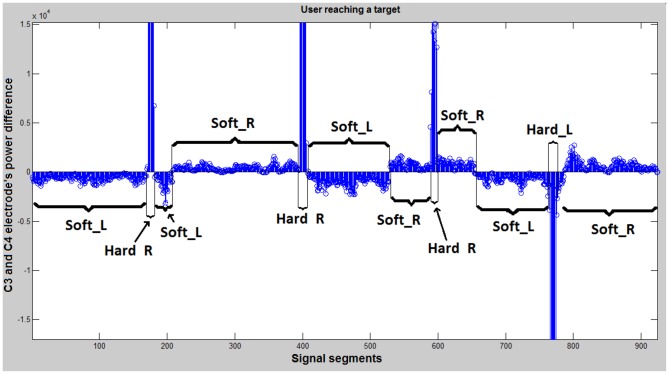
Online results from a user reaching a target (3rd step). SoftR corresponds to the low positive values, SoftL to the low negative values, HardR to the high positive values, and HardL to the high negative values. Relax task is not used (its power level is lower than SoftR and SoftL). Y-axis represents the PSD difference between C3 and C4. X-axis represents the number of analyzed data windows (each 50 ms a new data window is analyzed).

During this step, the user gets used to the 2-dimensional movement of the cursor and learns how to control it. Figure 12 shows the signals produced by a user when the two dimensional movement is being controlled. It clearly shows the difference between tasks SoftR, SoftL, HardR and HardL. Eight sessions are performed during this step and in each run, 10 targets appear. The user has 25 seconds to reach each target. Otherwise, the target counts as not reached and a new target appears.

### Protocol Summary

Each run is approximately four minutes long, taking one minute break between them. The total training time needed to control the two dimensions with the cursor can be computed as:

- Step 1: One start up run to get used to the kind of jaw movements the system requires.

- Step 2: Five runs where a model is defined for the user (selection of thresholds).

- Step 3: Eight final runs where the user learns how to control the two dimensional movement.

The training process takes approximately 70 minutes. After that, the user has an accurate control of the system and he/she is ready to start controlling the robotic arm as it is shown on the next section.

### Robot Control System

After the user is trained in the 2-dimensional control of the cursor, the system is going to be adapted to control the end-effector of a robotic arm on a 2-dimensional plane. Two different processes are running simultaneously to achieve this goal. A Matlab function is in charge of the processing and classification of signals and it also provides a simple graphical interface to help the users on their first contact with the robotic arm. A C++ program translates the classification results into control commands and sends them to the robotic arm.

#### Robotic Arm

**Figure 13:**
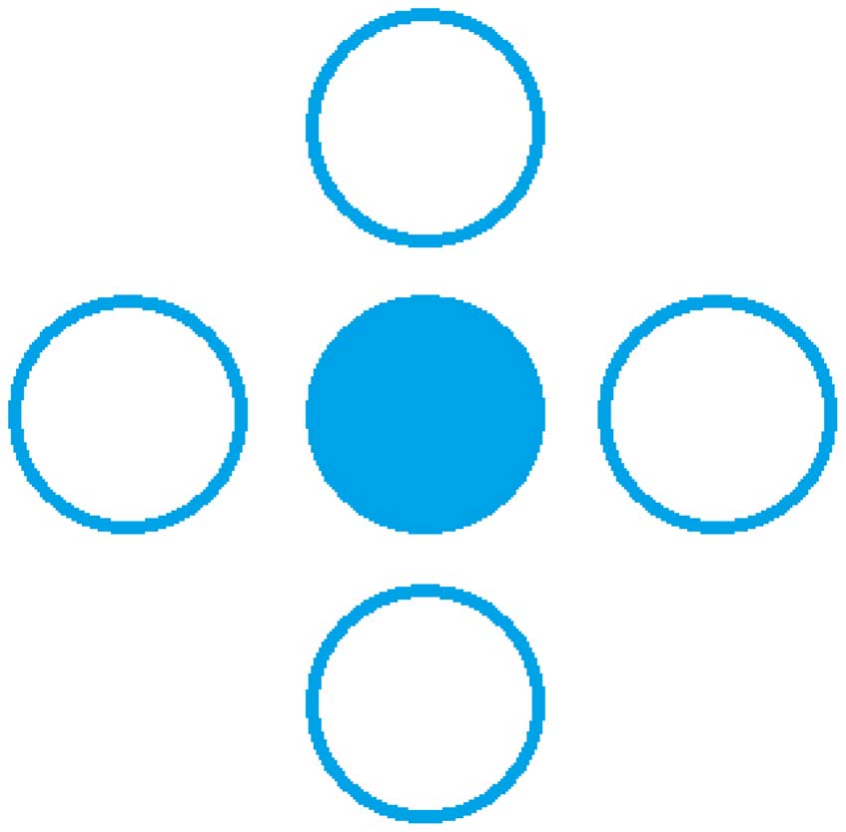
Robotic Arm Environment. It allows movements in a three dimensional workspace. Z-axis remains fixed during tests. The spots show the position of the 8 targets defined.

For the kind of movements wanted on this research, a 2.5D plotter robot may be a more suitable option but due to equipment already available in the research facility, the robotic arm used is the Fanuc LR Mate 200iB. It is a six degrees of freedom robot that can be moved in a three dimensional workspace. Figure 13 shows the robot appearance. The robotic arm is controlled using a C++ program through a local computer network. This program is used to send movement instructions to the LR Mate 200iB. It also receives information about the current position of the robot. Moreover, the C++ program runs a control panel whose main goal is to provide a set of simple instructions to control the interaction with the robot [1]. Through this panel, it is possible to connect and disconnect the robot. It also implements a function to send the robot to a home position and another function is in charge of sending movement instructions to the robot in real time according to the classification results provided by Matlab.

#### Graphical Interface

**Figure 14:**
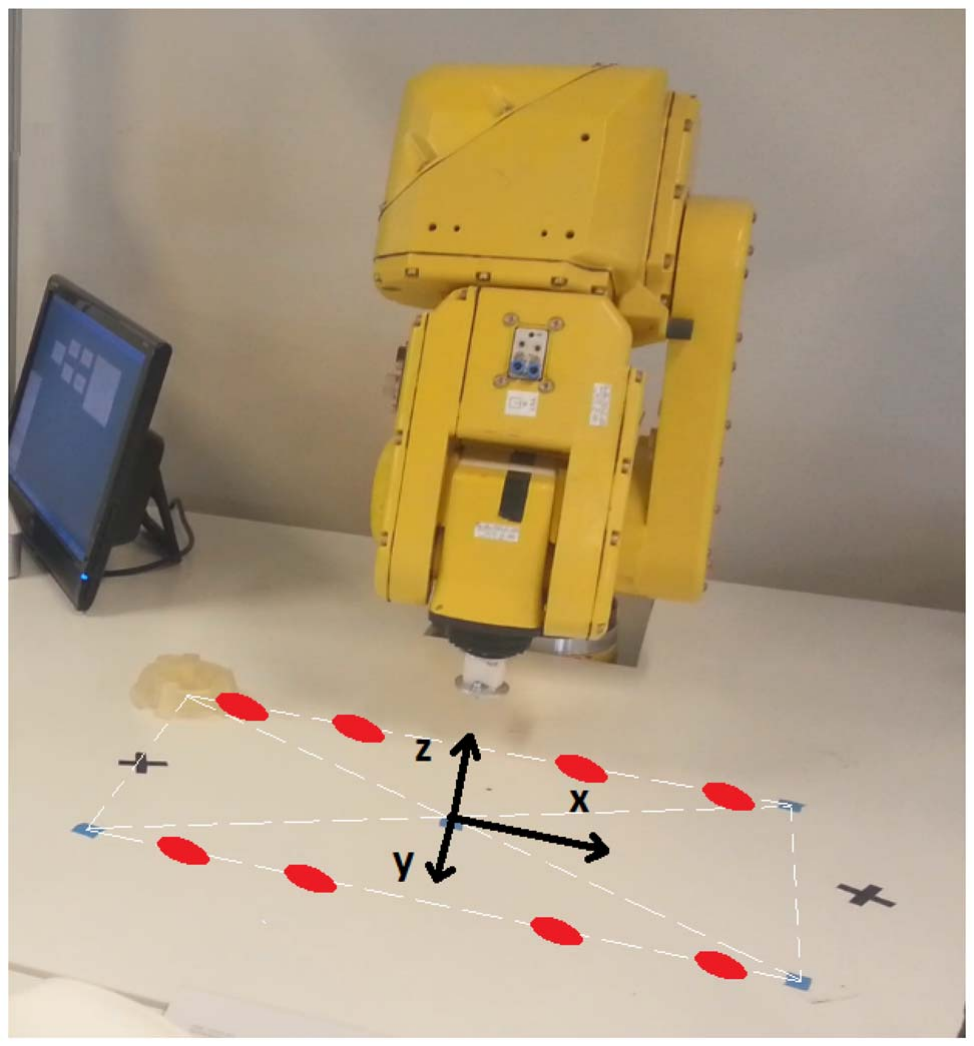
Visual feedback. The full circle represents the direction identified by the algorithm. The robotic arm moves according to the direction shown on this interface.

A Matlab-based application algorithm registers, processes and classifies the information recorded from the sensorimotor scalp areas. The C++ program uses the final data provided by Matlab in order to send movement instructions to the robotic arm. A communication system has been implemented to make possible the interaction between both processes. This communication introduces a small delay (less than 0.5 seconds) between the moment when the user executes the task and the moment when the robotic arm starts moving. For that reason, a graphical interface has been designed to provide visual feedback of each classified direction. This visual feedback uses the image shown in Figure 14. Each circle represents a direction in a two dimensional plane. This way, it is possible for a user to move the robotic arm to one of these directions. The full circle corresponding to the direction where the robot is moving gets coloured blue while the others remain empty. This interface is only used on the first run performed with the robotic arm. After that, the user gets used to the small delay and the interface can be removed.

#### Real Time Test

**Figure 15:**
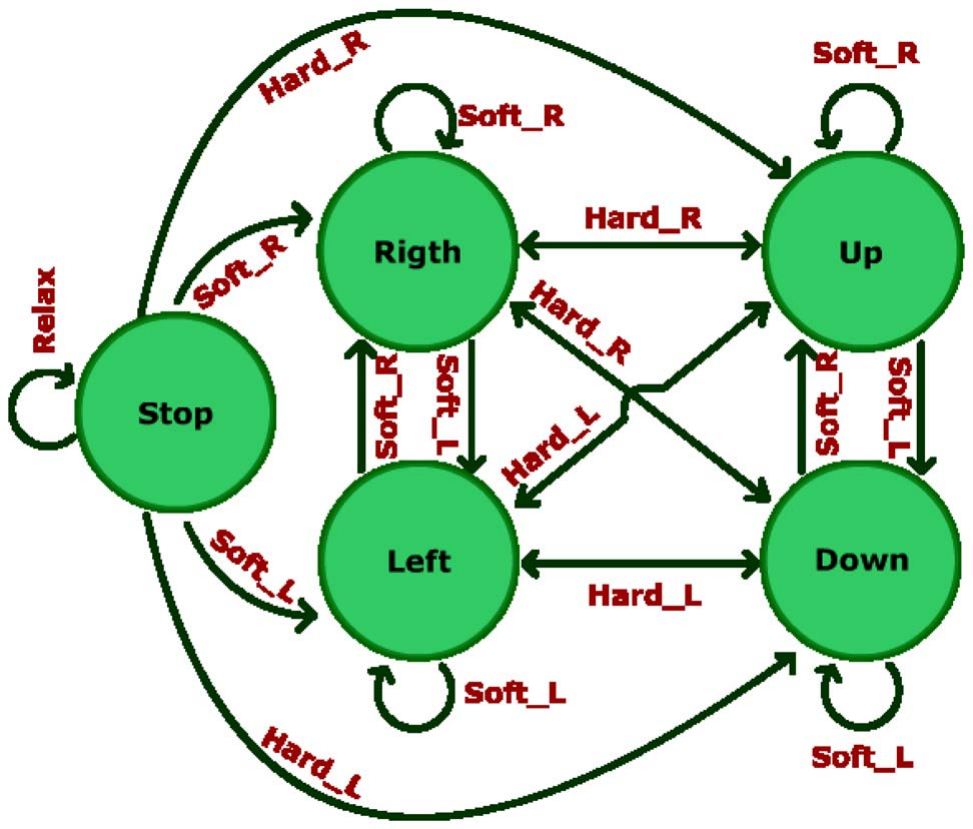
Robotic Arm State Machine. When a state changes, a continuous movement starts in the direction indicated by the current state.

Tests have been made over a two dimensional workspace. Z-axis is fixed and X-axis and Y-axis are restricted to a rectangle area DIN-A3 size. This way, the users who trained on a computer screen are familiar with the movement area. Figure 13 shows the mentioned workspace. As it can be seen, 8 targets have been placed on the workspace. The users were requested to reach these targets by controlling the robotic arm. Each user has performed nine runs. In the first run no target is requested. The user uses this run to get used to the system delay. The eight remaining runs are used to reach the eight possible targets shown on Figure 13. On each run, a different target is requested and the robotic arm movement stops when the target is reached. Robotic arm movement is controlled by the user through the five tasks previously described. In order to keep HardR and HardL as short duration tasks, a state machine (Figure 15) is designed. As it can be seen, the control of the two dimensional movement is similar to the one used on training step 3. The differences are due to the delay limitations introduced by the robot. Although the movement increment is detected by the system each 50 ms, the robotic arm cannot respond with such speed. For this reason, movement commands are sent only when a change of direction is detected in order to reduce the delay times. For instance, if a right movement state is detected, the robotic arm starts a right continuous movement until a new state is detected. At that moment, the current robot movement is stopped and a new movement begins.

### Results and Discussion

The system was tested with four healthy volunteers (capable of moving their jaws) with ages between 22 and 28 (24.25 ± 2.62) (three men with previous experience with this kind of tests and one woman without any, all of them right-handed). All the volunteers were sat while performing the experiment. They were told not to blink nor perform neck movements, except in rest periods indicated during the tests. The results presented on this work were obtained over a period of 3 months. The data of the cursor movement were registered in the first month and the robot arm control data in the third month. Human data presented in this article have been acquired under an experimental protocol approved by the ethics committee for experimental research of the Miguel Hernández University of Elche, Spain. Written consent according to the Helsinki declaration was obtained from each subject. Also the participants shown in Figure 3 and on referred videos have given written informed consent (as outlined in PLOS consent form) to publish these case details.

### Cursor Movement Results

During step 1, all users got used to the kind of clenching that the system is able to identify. They also achieved a successful threshold convergence during the 5 runs from step 2. On the third step, users 1, 2 and 4 noticed that tasks SoftR and SoftL are correctly detected by making a small jaw movement to the side the user wants to move the cursor without the need of clenching their teeth. User 3 kept clenching the jaw. All the users achieved tasks HardR and HardL by making a quick bite with the right or left side of the jaw. Table 1 shows the success and fail rate depending on the number of targets reached by a user in each run. User 2 is the system developer so it has more experience than the rest. As a consequence, he reached all the targets under the time limit. As it has been mentioned, the learning process takes place from runs 1 to 3. During these 3 runs, success rate experiences a huge improvement and after that it remains constant. The control that the user achieves after the third run is very similar to the control anyone can achieve using a joystick or other manual control as shown on video S1. In section Protocol Summary, the training time was estimated as 70 minutes, but by seeing these results, in 3 runs the control of the system is really close to its optimum. Thus it is reasonable to say that the training process could be reduced to 45 min. Table 2 shows the time efficiency in the reaching of targets where the time efficiency coefficient has been defined as follows: 
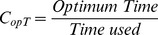
(3)


**Table 1:** Targets Reached.

	Run 1	Run 2	Run 3	Run 4	Run 5	Run 6	Run 7	Run 8
User 1	30	70	90	90	100	90	100	90
User 2	100	100	100	100	100	100	100	100
User 3	40	80	90	90	100	100	80	100
User 4	80	80	100	100	90	90	100	100

Percentage (%) of targets reached by the user on each run.

**Table 2:** Coefficient of Spent Time to Reach the Target with the Cursor.

		1	2	3	4	5	6	7	8	Avg
	1	0.636	0.688	0.815	0.790	0.784	0.824	0.733	0.801	0.759
	2	0.868	0.915	0.891	0.927	0.856	0.802	0.900	0.914	0.884
Users										
	3	0.657	0.768	0.812	0.740	0.739	0.791	0.825	0.784	0.765
	4	0.834	0.742	0.838	0.814	0.727	0.801	0.767	0.820	0.793
	Avg	0.749	0.778	0.839	0.818	0.777	0.804	0.806	0.830	**0.800**

The values show the 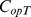 for each user to reach every target.

The *time used* is the time spent for the user to reach the target, and *optimum time* is the time needed to reach the target if the cursor is controlled manually. To obtain the optimum time, an algorithm that allows a subject to control the cursor by using the key arrows is implemented. All users were asked to reach each target using this manual control. The *optimum time* for each target is calculated as the average of the time employed by all users using this manual control (this average presents a very small deviation meaning that the optimum times are very similar between users). The non-reached targets were not computed to obtain these results. 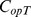 is in average 0.8 throughout the 8 runs for all subjects. In order to prove the efficiency of this system, the average value of the obtained 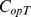 is compared with the average 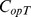 of a BMI system based on motor imagery tasks and a system based in electrooculography (which usually provides better results than motor imagery systems). The optimum time is calculated on these systems according to the methodology previously explained. Analyzing the results from [27], [28] (which are previous works done by our group in a similar environment with a hieralchical motor imagery BMI system and a electrooculographic system) the average 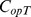 obtained in the BMI system is 0.033, which is 30.30 times worse than the optimum time, and the average 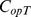 obtained in the electrooculographic system is 0.588, which is 1.7 times worse than the optimum time. Using our system to move a cursor on a screen, the average 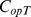 for all runs and users is 0.8, which is 24.24 times quicker than the motor imagery BMI system (0.033) and 1.36 times quicker than the electrooculographic system (0.588).

### Robotic Arm Movement Trials Results

After the training section, users are ready to control the robotic arm in two dimensions. They are requested to reach eight different targets. Time required to reach each target is measured. Table 3 shows the values of 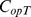 obtained by the users to reach each target referred to the minimum time needed by the robot to reach them (manually controlled) under the same conditions. The strategy used in the previous section was applied here to obtain the values of the *optimum time*. All users were also requested to reach the targets using the arrow keys. In this case, there is no improvement along runs due to the similarities between the cursor movement system (Training section) and the robot movement system. Results are also compared with the needed time percentages to reach targets using a system based on motor imagery tasks seen on the last section (0.033). This time, the improvement is, in average, 0.818, which is 24.78 times better than a motor imagery BMI system and 1.4 times better than the electrooculographic system. On videos S2 and S3 are shown 2 subjects who have completed the training in a free movement test reaching a target with and without obstacles in the workspace.

**Table 3:** Robotic Arm Time Percentage.

		1	2	3	4	5	6	7	8	Avg
	1	0.856	0.768	0.931	0.894	0.680	0.904	0.833	0.816	0.835
	2	0.904	0.955	0.707	0.669	0.904	0.899	0.855	0.859	0.844
Users										
	3	0.879	0.990	0.837	0.946	0.837	0.938	0.821	0.911	0.895
	4	0.994	0.837	0.890	0.755	0.788	0.764	0.988	0.622	0.830
	Avg	0.908	0.890	0.841	0.816	0.802	0.876	0.874	0.802	**0.851**

The values shown indicate the 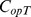 for each user to reach every target.

### System Limitations

The number of states able to be detected on this system is limited by the behavior of the measured signals. For soft clenching, the PSD increment can be easily controlled by the user, but when the clench is higher, the PSD behaves similar to an exponential function, increasing considerably the control difficulty. Also, thresholds have been defined in order to benefit tasks SoftR, SoftL, HardR and HardL, but making hard for some users the control of the Relax state. This point should be further studied in future works.

## Conclusion

A supplementary application for a BMI system has been designed. A very similar system could also be implemented placing a couple of electromyographic electrodes on both cheeks or placing pressure sensors on the teeth. However, the goal of this research is to use the electrodes of a BMI system in order to implement a supplementary system based on the skill of a user to control EMG signals produced by clenching different areas of the jaw. Results prove that the control acquired by users who can move their jaws is close to the control that a healthy user can acquire using a joystick or the movement arrows of a keyboard.

**Figure 16:**
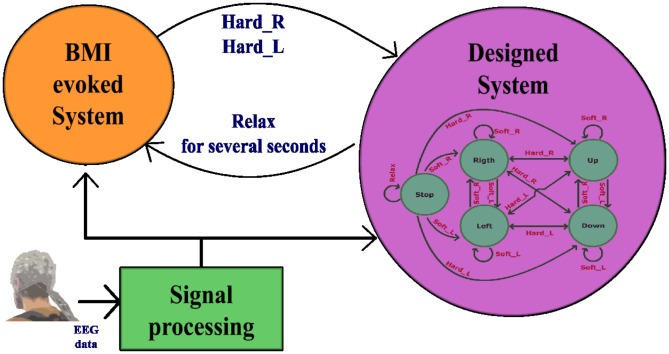
Block interconnection diagram. The supplementary system is activated with tasks HardR or HardL, while the control is returned to the BMI system when the user stays on the Relax state for several seconds.

On the basis of this study, the architecture shown on Figure 16 is proposed as a future work. A BMI system would work while the jaw is relaxed. At the moment the user wants to alternate to jaw control, a quick bite (tasks HardR or HardL) would activate the jaw control algorithm. The change to the BMI system should be produced when the user remains several consecutive seconds on the Relax state. Our supplementary system should be complemented with an appropriate BMI system, i.e. a menu application that allows a patient to select the rehabilitation strategy desired while a BMI system measures the mental state of the patient in order to evaluate how the selected strategy affects the mental workload of the patient. It is also proposed its use in combination with a BMI system previously developed by our group [24], where visual evoked potentials were used to control an internet browser, allowing a user to write and move the cursor on the screen. The BMI system provides a quick and fluid writing but the cursor control can be improved by using the supplementary system designed. On the same work, the BMI system is used to control a robot arm in 3 dimensions. For that purpose, the user first selects the movement plane and then, a 2-dimensional control also based on evoked potentials is used. Using the 2-dimensional control implemented in the current work, it would be possible to improve the performance of the 3-dimensional control described in [24], by combining the plane selection algorithm of the evoked BMI system and the 2-dimensional control implemented in our supplementary system. As a conclusion, this supplementary system allows us to implement a control system in combination with a BMI system using the same set of sensors. This system is oriented to help people who suffer from motor disabilities which deprive them from moving their arms or legs but still have mobility on the jaw. In the future, this system should be tested on this kind of patients. The system might be also adapted for 3-dimensional movement by alternating between three space axes instead of two. Another research line would be to compare a third electrode from a central position (like Cz or CPz) with C3 and C4 in order to measure separately the level of clench from left and right areas of the jaw so both dimensions, X and Y, would be simultaneously controlled.

## Supporting Information

Video S1 **User 2 controlling a cursor on a screen.**
(MP4)

Video S2 **User 2 controlling the robotic arm.**
(MP4)

Video S3 **User 1 controlling the robotic arm.**
(MP4)
