# Correction: A Supplementary System for a Brain-Machine Interface Based on Jaw Artifacts for the Bidimensional Control of a Robotic Arm

**DOI:** 10.1371/journal.pone.0118257

**Published:** 2015-02-06

**Authors:** The PLOS ONE Staff

There are errors in the Funding section. The correct funding information is as follows: This research has been funded by the Spanish Ministry of Economy and Competitiveness as part of the Brain2motion project—Development of a Multimodal Brain-Neural Interface to Control an Exoskeletal: Neuroprosthesis Hybrid Robotic System for the Upper Limb (DPI2011-27022-C02-01). The funders had no role in study design, data collection and analysis, decision to publish, or preparation of the manuscript.
